# Impulsivity and comorbid traits: a multi-step approach for finding putative responsible microRNAs in the amygdala

**DOI:** 10.3389/fnins.2014.00389

**Published:** 2014-12-11

**Authors:** Andrzej Z. Pietrzykowski, Sabine Spijker

**Affiliations:** ^1^Department of Animal Sciences, Rutgers UniversityNew Brunswick, NJ, USA; ^2^Department of Genetics, Rutgers UniversityPiscataway, NJ, USA; ^3^Department of Molecular and Cellular Neurobiology, Center for Neurogenomics and Cognitive Research, Neuroscience Campus Amsterdam, VU UniversityAmsterdam, Netherlands

**Keywords:** impulsive action, miR-28a, miR-340, miR-219a, miR-491, miR-346, recombinant inbred strain, bioinformatics

## Abstract

Malfunction of synaptic plasticity in different brain regions, including the amygdala plays a role in impulse control deficits that are characteristics of several psychiatric disorders, such as ADHD, schizophrenia, depression and addiction. Previously, we discovered a locus for impulsivity (*Impu1*) containing the neuregulin 3 (Nrg3) gene, of which the level of expression determines levels of inhibitory control. MicroRNAs (miRNAs) are potent regulators of gene expression, and have recently emerged as important factors contributing to the development of psychiatric disorders. However, their role in impulsivity, as well as control of Nrg3 expression or malfunction of the amygdala, is not well established. Here, we used the GeneNetwork database of BXD mice to search for correlated traits with impulsivity using an overrepresentation analysis to filter for biologically meaningful traits. We determined that inhibitory control was significantly correlated with expression of miR-190b, -28a, -340, -219a, and -491 in the amygdala, and that the overrepresented correlated traits showed a specific pattern of coregulation with these miRNAs. A bioinformatics analysis identified that miR-190b, by targeting an Nrg3-related network, could affect synaptic plasticity in the amygdala, targeting bot impulsive and compulsive traits. Moreover, miR-28a, -340, -219a, and possibly -491 could act on synaptic function by determining the balance between neuronal outgrowth and differentiation. We propose that these miRNAs are attractive candidates of regulation of amygdala synaptic plasticity, possibly during development but also in maintaining the impulsive phenotype. These results can help us to better understand mechanisms of synaptic dysregulation in psychiatric disorders.

## Introduction

Impulsivity is a personality trait that often occurs in psychiatric disorders. It is one of the hallmarks of attention deficit hyperactivity disorder (ADHD)(Barkley, 1997), substance abuse, bipolar disorder and schizophrenia (McElroy et al., 1996; Koob and Le Moal, 1997; Nestor, 2002; Koob and Volkow, 2010; Lombardo et al., 2012). Increase in impulsivity has been also reported in brain injuries (Bechara and Van der Linden, 2005), in Parkinson's disease secondary to dopaminergic therapy (Wolters et al., 2008; Callesen et al., 2013), or Alzheimer's disease (Rochat et al., 2008). Impulsivity, or the lack of impulse control, is a multifactorial construct (Evenden, 1999) that involves both impulsive choice, and impulsive action; the latter also referred to as the absence of inhibitory control. High impulsive choice is measured by an increased preference for an immediate small over a larger but delayed reward that is more beneficial for the individual. On the other hand, inhibitory control is the ability to inhibit or hold back a prepotent response. Thus, a high level of impulsive action is characterized by poorly conceived, prematurely expressed, unduly risky, or inappropriate actions that often result in undesirable consequences in the long-term. Emotional responses typical for impulsive behavior are orchestrated by several neuronal structures, among which the amygdala is in part responsible for impulsive choice (Gupta et al., 2011) and impulsive action (Depue et al., 2014), as well as for the compulsive component, like addiction (Koob, 2009; Koob and Volkow, 2010). The comorbid nature of impulsivity reflects a multifactorial etiology of psychiatric disorders, and knowledge of its full biological underpinning remains scarce.

Current models explaining psychiatric illnesses have abandoned simple neurotransmitter models, and rather hold disruptions across whole cellular networks responsible. In this view, amongst other factors contributing, microRNAs (miRNAs), which could modulate expression of network of genes and proteins, have the potential to bridge the current gap in our knowledge between the treatment of these debilitating disorders and the underlying neurobiology. After the discovery of the first miRNA in the early nineties (Lee et al., 1993; Wightman et al., 1993), these 18–25 nucleotides long, single-stranded, non-coding RNA species, have emerged as important regulators of post-transcriptional gene expression (Ambros and Lee, 2004; Landgraf et al., 2007; Friedman et al., 2009), including constituents of synaptic structures within neuronal circuits (Siegel et al., 2011). Typically, a miRNA can target multiple mRNAs, by binding to its specific miRNA binding site located within 3′-untranslated region (3′-UTR) of each targeted mRNA (Farh et al., 2005; Lewis et al., 2005). This way a single miRNA can regulate activity of multiple members of a complex biological network. Although miRNAs have been linked recently to the development of psychiatric disorders (Im and Kenny, 2012; Xu et al., 2012; Nestler, 2014), neurodegenerative diseases (Hébert and De Strooper, 2009), and actions of drugs of abuse and alcohol (Pietrzykowski, 2010), a role for miRNAs in impulsivity is not well established. Thus, far, only some indirect evidence suggests that miRNAs may play a role in this trait. An association between two single nucleotide polymorphisms (SNPs) located within the miR-96 gene and attention deficit/hyperactivity disorder (ADHD) was reported (Sánchez-Mora et al., 2013). Additionally, an association between trait impulsivity using self-reporting measures and SNPs located in a genomic region encoding the 3′-UTR of the SNAP-25 mRNAs that contain the miR-641 binding site was described (Németh et al., 2013).

Recently, we used a forward genetic screen in the recombinant inbred mouse set of BXDs (Loos et al., 2014), to question what factors control levels of inhibitory control. These mice form a genetic reference population with a fixed genome allowing assessment of genetic covariance in terms of behavior and modulation of transcriptional activity (Plomin et al., 1991; Williams et al., 2001; Peirce et al., 2004; Chesler et al., 2005). We measured inhibitory control by the 5-choice serial reaction time task (5CSRTT), a task with face validity to the human continuous performance task, in which attention parameters and motor impulsivity can be assessed (Cole and Robbins, 1989; Puumala et al., 1996; Humby et al., 2005). We identified the impulsivity locus *(Impu1)* located on chromosome 14 around marker rs6197032, at 34.5–41.4 Mb (build 37, mm9) (McElroy et al., 1996; Lombardo et al., 2012; Loos et al., 2014). This locus harbors Neuregulin3 (Nrg3), a member of the neuregulin family, which is important for nervous system development as well as schizophrenia (Sachs et al., 2000; Barros et al., 2009). The fact that the C57BL/6J allele contributed to increased impulsivity, that C57BL/6J mice showed increased *Nrg3* expression in the medial prefrontal cortex (mPFC), a region important for impulsivity, and that Neuregulins play an important role in central nervous system function and neuropsychiatric diseases, culminated in testing the effect of Nrg3 overexpression in the mPFC on impulsivity. As hypothesized, higher levels of Nrg3 resulted in higher levels of impulsive action, whereas loss of Nrg3 decreased impulsive responding (Palanza, 2001; Krishnan and Nestler, 2011; Loos et al., 2014; Zhu et al., 2014). Together this showed that regulating Nrg3 levels is key to impulsivity, in which miRNAs could play an important role.

With the realization that specific disease symptoms could occur in several diseases, analysis of intermediate traits, known as endophenotypes (Almasy and Blangero, 2001; de Geus et al., 2001; Loos et al., 2009), soon became a popular strategy to find genetic and molecular underpinning of complex phenotypes. This strategy has the advantage that animal models, harboring a fraction of the complexity of the disease, could contribute to solve the puzzle. Co-occurrence of several of these endophenotypes in a model could point to a shared molecular mechanism. In this study we used a bioinformatics approach (Robbins et al., 2012; Mulligan et al., 2013) to question what behavioral effects are comorbid with the impulsivity trait (Loos et al., 2014), and what miRNAs could be driving levels of impulsivity. Therefore, we performed a comprehensive analysis to first select traits that are overrepresented with impulsivity, and second to select microRNAs of which the expression in amygdala is associated with impulsivity, using GeneNetwork data and several bioinformatic tools. We selected microRNAs based on three criteria; (1) their location within the *Impu1* locus, (2) targeting of the *Nrg3* mRNA, and (3) a correlated expression with impulsivity and comorbid traits. Together, we observed that miRNAs miR-190b, miR-28a, -219a, -340, and -491-5p may contribute to synaptic rearrangements and plasticity in the “impulsive” amygdala.

## Methodology

### Genenetwork correlation analysis

From the GeneNetwork database (http://www.genenetwork.org) (Rosen et al., 2007), we selected behavioral traits correlated with motor impulsivity (number of premature pokes; trait #16311) as measured in the 5CSRTT (Loos et al., 2014) and performed the analysis in a two-step fashion: (1) we ranked correlations using Spearman rank test with n-number larger than 15 overlapping strains, as many traits in the database are from relatively small cohorts, and with corrected *P*-value < 0.01; and (2) we performed a trait overrepresentation test using key word searches, in which significantly correlated traits should be overrepresented in the GeneNetwork database. Key words representing the trait of interest were selected to preferably yield ~50–100 traits upon searching the GeneNetwork database, in order to get evenly sized groups based on this search.

For miRNA correlating with impulsivity, we focused on the amygdala because of its importance in emotional regulation, and because miRNA expression in BXD mice is well documented in the GeneNetwork database (http://www.genenetwork.org/dbdoc/INIA_AmgCoh_0311.html) with expression data available for 50 BXD strains. Significant correlations were calculated for traits with ≥25 strains in overlap. Correlation between traits or between traits and amygdala miRNAs is shown as the correlation coefficient (rho; Pearson product moment) with two-sided *P*-value indicated.

### microRNA validation

Expression of miRNAs used for correlation analysis was based on Affymetrix probes (**Table 3**) of the GeneNetwork amygdala database (INIA Amygdala Cohort Affy MoGene 1.0ST (March 11 RMA). We validated each probe set specificity in detecting miRNA expression by alignment of probe sequences with the most current mouse genome browser and establishing their locations to miRNA genes. Location of these genes and sequence alignment of probe sets to these genes was further validated by miRBase, a free biological database that acts as an archive of all miRNA sequences and annotations (Griffiths-Jones, 2006; Griffiths-Jones et al., 2006), which also provides naming standards of microRNAs. Since recently miRNA nomenclature has changed (Arora et al., 2013), we matched GeneNetwork miRNA names with the most current miRNA names and used them throughout the paper (**Table 3**). Note also that GeneNetwork and miRBase are using different mouse genome assemblies, each providing different genomic coordinates for microRNA genes. Therefore, we used the NCBI Remap tool (http://www.ncbi.nlm.nih.gov/genome/tools/remap) to convert and match positions between assembly versions. Also, presence of SNPs in the regions targeted by probes can affect their efficacy, and may lead to false gene expression level results. We verified, using GeneNetwork variant browser, lack of SNPs in the genomic sequences targeted by these probes in both, C57Bl/6J and DBA2/J mice (**Table 3**).

### Targetscan

We used TargetScanMouse (Release 6.2) to predict mouse miR-190b-5p targets. Among many miRNA target prediction software TargetScan seems to be very reliable (Friedman et al., 2009), and it uses two prediction scores. The aPCT score (a positive score) is based on the probability of aggregated preferentially conserved targeting, which is an indicator that a site is conserved due to selective maintenance of miRNA targeting rather than by chance. The total context (TC) score (a negative score) provides predictions based on four features of the putative microRNA-binding site: length of its complementarity to the microRNA's seed region (7-mer or 8-mer), position of the complementarity (3′-end), flanking of the binding site by adenosines, and a position within the 3′-UTR. To ensure high probability of interactions between miR-190b-5p and its targets and avoid false-positives we applied stringent criteria using simultaneously both scores (aPCT > 0.2; CT < -0.07) thereby selecting the top ~33% of targets (34 out of 103 total targets) with the highest aPCT score and the lowest TC score.

### GeneMania

We used GeneMania to predict the miR-190b/Nrg3 interactive network. GeneMania is a freely available web interface designed to determine molecular interactions based on a set of input genes in different species (Warde-Farley et al., 2010). We originally input all 34 targets of miR-190b-5p using network weighting as “equal as determined by the network” (see **Figures 4A,C**), or using Gene Ontology (GO)-based weighting for “biological terms” (see **Figures 4B,D**) to maximize prediction of connectivity between all input genes. GeneMania provides eight interaction categories (co-expression, co-localization, physical interaction, genetic interaction, shared protein domains, pathway co-participation, predicted relationship and other) with ten to a hundred new genes shown. We restricted our output data to only ten new genes and three categories (co-expression, co-localization, physical interaction). We chose these three categories because they describe the most direct interactions between molecules. This approach allowed us to obtain a small network of highly interconnected genes with strong interactions centered on Nrg3. In a second round, 3 targets, and 4 related genes were used as input.

In addition, we used GeneMania to perform the functional enrichment analysis based on Gene Ontology (GO) terms augmented among genes in the network. Only biological terms with *P* < 0.05 after correction for the false discovery rate (FDR) were considered to be enriched. We further used DAVID (The Database for Annotation, Visualization and Integrated Discovery) (Huang et al., 2007) and GeneCards (Rebhan et al., 1997) to validate GeneMania results describing association of members of the Nrg3-network with specific functional terms.

### mirPath

DIANA mirPath (Maragkakis et al., 2009) is a freely available web interface designated by DIANA (DNA Intelligent Analysis) Laboratory to estimate the impact miRNA effects on biological pathways. Both the effect of a single miRNA and the combinatorial effects of multiple miRNAs are available. After performing an enrichment analysis mirPath marks positions of miRNA-targeted genes in biological pathways provided by Kyoto Encyclopedia of Genes and Genomes (KEGG) (Kanehisa and Goto, 2000). The KEGG Pathway database is the most comprehensive resource of interactions of gene products and provides wiring diagrams of interaction networks. DIANA mirPath output consists of KEGG pathways sorted according to *P* value after correction for the false discovery rate (FDR, Hochberg and Benjamini, 1990). Only pathways with *P* < 0.05 were considered to be enriched. Lower *P*-value indicates bigger biological impact of miRNA(s) on that pathway.

## Results

### Correlations of impulsivity with other behavioral traits

**Figure 1:**
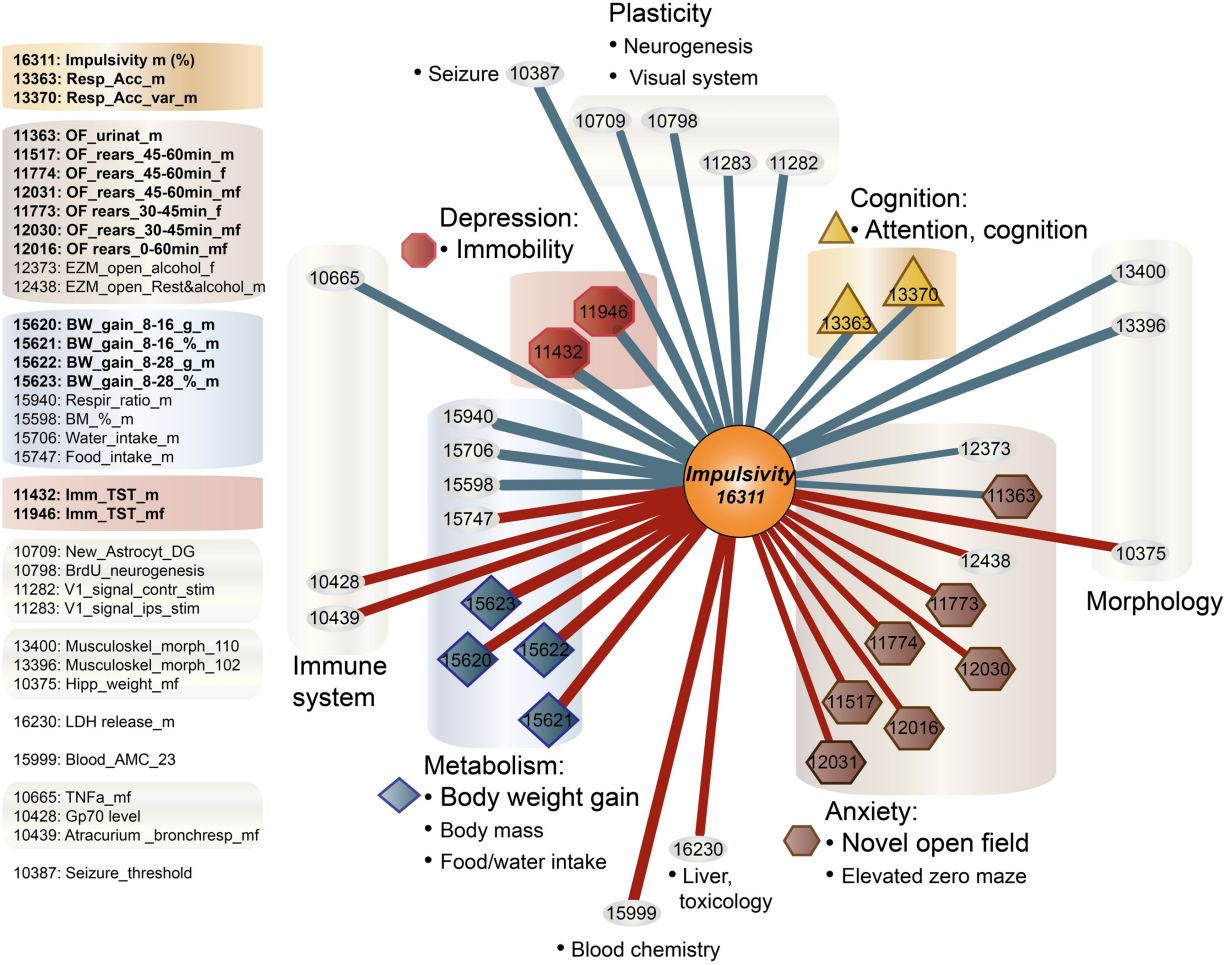
**Correlation of traits with impulsivity in BXD strains**. Individual behavioral traits and their compound traits (colored) are shown. Traits number IDs are taken from the GeneNetwork records. The impulsivity trait (ID#:16311) is placed in the center of the figure. Color of lines indicates the direction of the correlation (blue, negative; red, positive), and line thickness corresponds to the size of the Spearman rank correlation (thicker line means stronger correlation).

Impulsivity, measured as the lack of inhibitory control in 5CSRTT (Loos et al., 2012, 2014), is a characteristic of many psychiatric diseases (e.g., ADHD, schizophrenia, depression, addiction), each represented by complex interactions of several traits. To understand the role of miRNAs in impulsivity, we first assessed whether impulsive behavior would correlate with other phenotypic traits, as this could be an indication that the genetic make-up of BXD strains controlling impulsivity play a role in other traits as well. Using the GeneNetwork database, we performed the analysis in a two-step fashion: (1) we ranked correlations using Spearman rank test with n-numbers larger than 15 overlapping strains, and with *P*-values < 0.01; and (2) we performed a trait overrepresentation test using key word searches, in which significantly correlated traits should be overrepresented in the GeneNetwork database. This approach should prevent finding of a correlation by pure chance, albeit that there still could be a bias toward studies with more in depth phenotyping. In total, we selected 34 traits (Table 1, Figure 1). From these 34 traits, 17 compound traits were selected using a Fischer's exact test (Table 2, Figure 1). Three of these traits (“Attention/Cognition,” “Depression/Immobility,” “Metabolism/Body weight gain”) were overrepresented showing a significant correlation (*P* < 0.05) with impulsivity, whereas one trait (“Anxiety/Novel open field”) showed a trend (*P* < 0.10) toward overrepresentation. Although classical parameters of an open field relate to anxiety, the correlated traits were mostly related to rearing behavior.

**Table 1:**
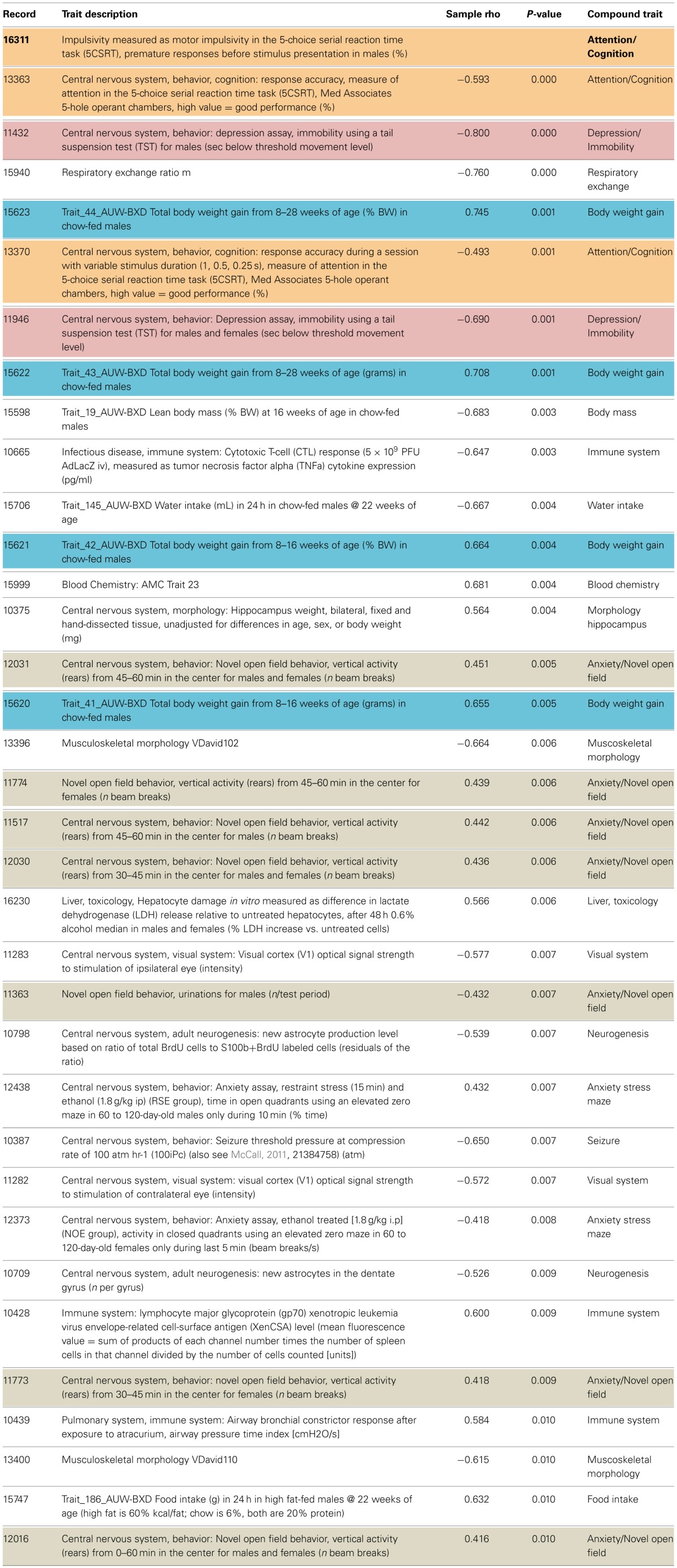
**Correlation of phenotypic traits with impulsivity in BXD mouse strains**.

The impulsivity trait (16311; bold) was correlated with all available phenotypic traits using GeneNetwork. Traits of the highest significant correlations (P < 0.001) for n = 15 BXD strains are displayed with their Spearman rank correlation coefficient (Sample rho) sorted by the P-value. Compound traits are color-coded when overrepresented (see Table 2).

**Table 2:** **Selection of overrepresented compound traits correlated with the impulsivity trait (16311)**.

**Compound trait**	**#Correlated traits**	**Total traits in GeneNetwork**	**Representation ratio**	***P*-value**
**Depression/Immobility**	**2**	**6**	**18.14**	**0.0096**
**Attention, cognition**	**2**	**9**	**12.09**	**0.0181**
**Metabolism/Body weight gain**	**4**	**57**	**3.82**	**0.0314**
**Blood chemistry**	**1**	**345**	**0.16**	**0.0330**
**Immune system**	**3**	**499**	**0.33**	**0.0443**
*Anxiety/Novel open field*	*7*	*179*	*2.13*	*0.0942*
Neurogenesis	2	51	2.13	0.2186
Respiratory exchange	1	18	3.02	0.3045
Food intake	1	18	3.02	0.3045
Muscoskeletal morphology	2	276	0.39	0.3076
Seizure	1	29	1.88	0.4354
Body mass	1	30	1.81	0.4460
Hippocampal morphology	1	48	1.13	0.6050
Anxiety stress maze	2	76	1.43	0.6625
Liver toxicology	1	76	0.72	1.0000
Water intake	1	66	0.82	1.0000
Visual system	2	67	1.62	1.0000
Total	34	1850		

From 34 selected traits (see Table 1), 17 compound traits were generated based on key words analysis. The compound traits were sorted on P-value. A two-sided Fischer's exact test was used to determine significance of over- or underrepresented traits (P < 0.05, bold), or a presence of a correlation trend (P < 0.10, italics) using the total number of traits found in the GeneNetwork database. Representation ratio indicates the fold of the over- (black) or under- (gray) represented traits compared with the original query.

### Selection and correlation of microRNAs with impulsivity and compound traits

The amygdala is involved in emotional regulation of impulsive behavior, and miRNA expression in this brain region of the BXD mice is well documented in the GeneNetwork database. Therefore, we used this miRNA expression dataset to determine which miRNA correlates with impulsivity and its overrepresented traits. In addition, two traits (#15598, #15747) were added to the “Body weight gain” compound trait, as they relate to body weight and food intake, respectively. In our analysis we used a comprehensive, three-prong approach of miRNA selection.

**Figure 2:**
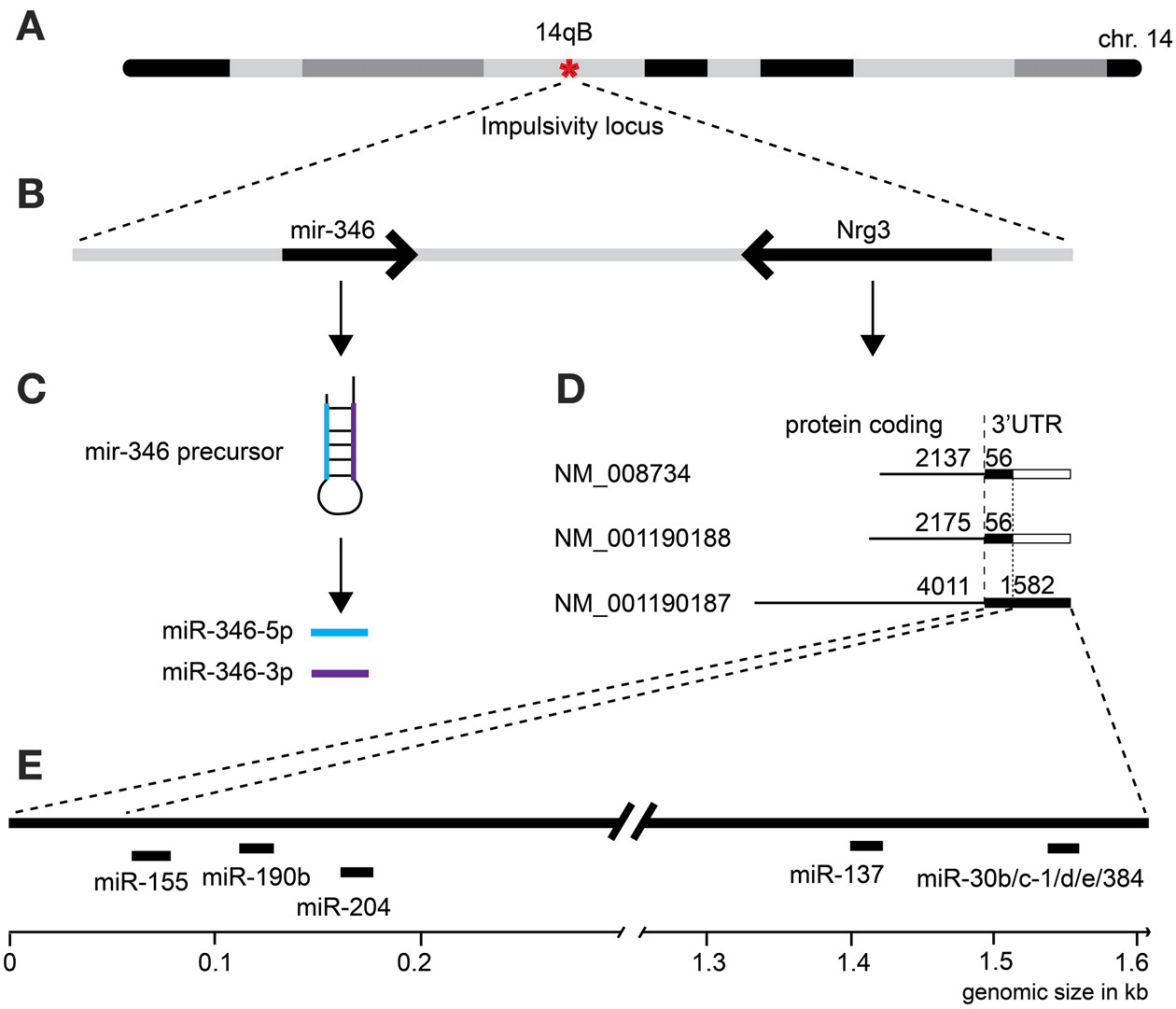
**Selection of microRNAs and their relationship with the impulsivity locus. (A)** The impulsivity locus is located on a short arm of mouse chromosome 14; **(B)** both, the mir-346 gene and the *Nrg3* gene are located within the locus; **(C)** the product of the mir-346 gene is a double-stranded RNA hairpin-like microRNA precursor, which gives rise to two mature miRNAs: miR-346-5p and miR-346-3p; **(D)** the *Nrg3* gene produces three mRNAs of different length and composition; **(E)** only the longest *Nrg3* mRNA (NM_001190187) contains miRNA binding sites.

First, we selected a miRNA located within a recently established impulsivity locus located on chromosome 14 in mice (Loos et al., 2014) (Figure 2A). Despite a substantial size of this locus (6.9 Mb), it contains only a single miRNA gene (chr14:34,894,609–34,894,706) called mir-346 (Figure 2B). A product of the mir-346 gene is a hairpin-shaped mir-346 precursor, which gives rise to two mature miRNAs: miR-346-5p and miR-346-3p (Figure 2C), of which the former is a predominant form. Expression of the miR-346-5p miRNA in BXD mice was not correlated with impulsivity (Table 3), and therefore was not included into further analyses.

**Table 3:**
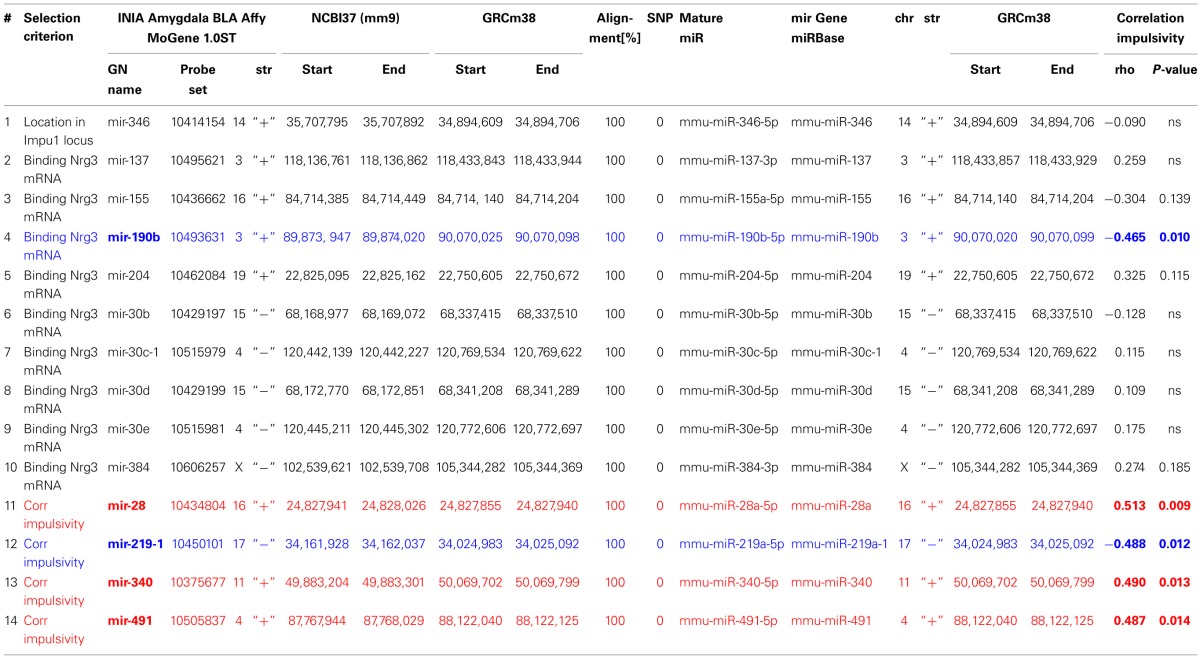
**Correlation of impulsivity with selected microRNAs expressed in amygdala**.

The miRNAs were selected based on three criteria described. The miRNA GeneNetwork (GN) name, Affymetrix Mouse Gene 1.0 ST probe number used to determine miRNA expression in amygdala of BXD mice, alignment in NCBI37 and GRCm38, miRNA full names comply with the most recent miRNA naming terminology, SNPs, sample rho and P-value of Pearson correlation with the impulsivity trait are shown. Significantly correlated miRNA are color-coded: negatively correlated miRNAs are blue, while positively correlated microRNAs are red. P < 0.05 indicates significant correlation (bold), P-values < 0.2 are indicated, otherwise non-significant (ns).

**Figure 3:**
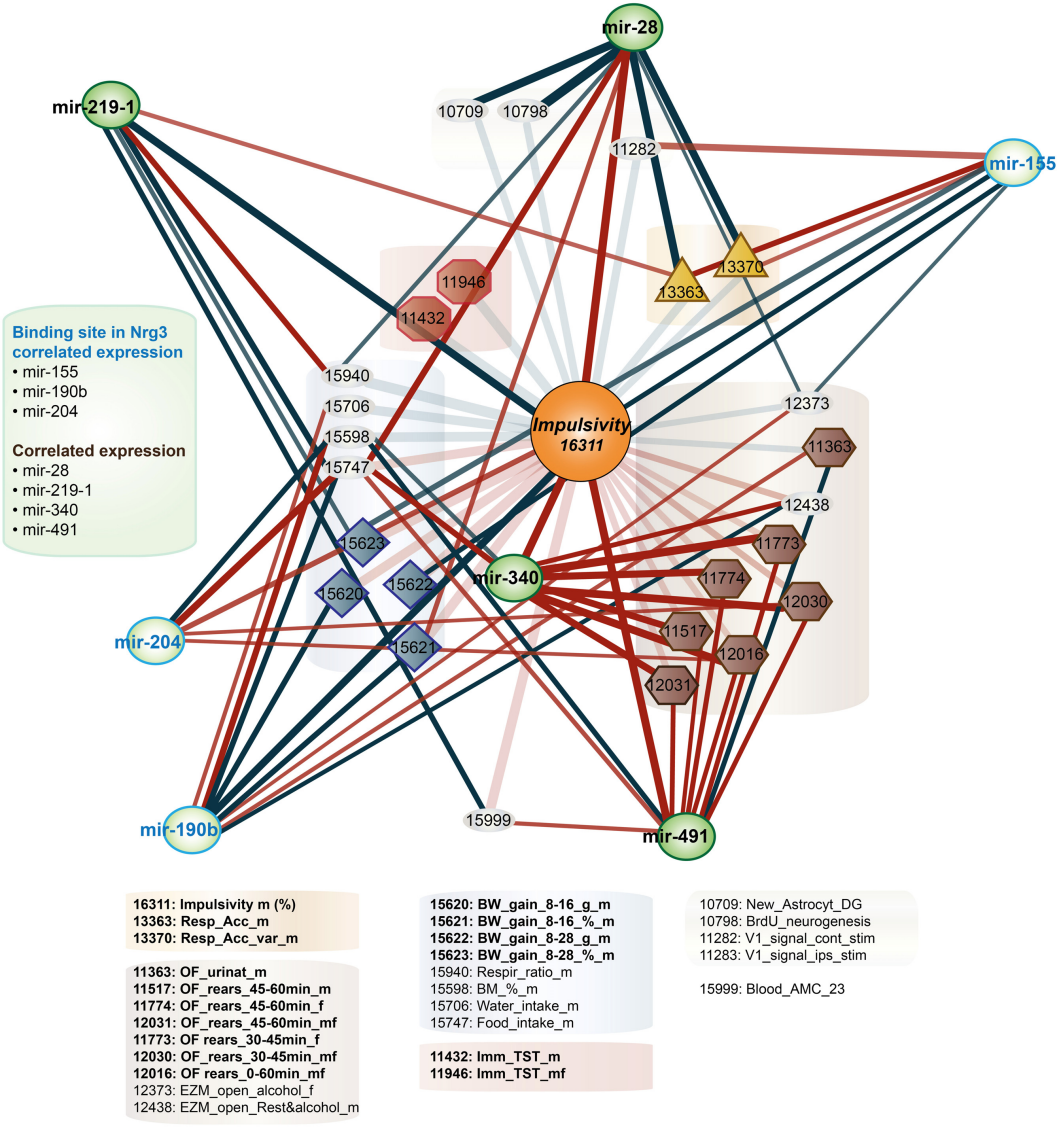
**Impulsivity-related microRNA expression network in amygdala**. Amygdala miRNAs (green circles) of which the expression is correlated with impulsivity (lack of inhibitory control) (Table 3) are shown. Correlation of miRNAs with impulsivity and compound traits are shown as lines. Colors of lines indicate the direction of the correlation (blue, negative; red, positive), intensity of the color depicts *P*-value of Pearson correlation (one sided: dark, *P* < 0.05; light, *P* < 0.10) and line thickness corresponds to the size of the Pearson rank correlation (thicker line means stronger correlation). This network is overlaid on the impulsivity correlation trait network from Figure 1 (faded colors) to show relationship of traits and miRNAs. For the GeneNetwork IDs of individual traits see Table 1.

Second, due to our previous observation of a significant association between *Nrg3* gene expression and the lack of inhibitory control, or impulsive action (Loos et al., 2014), we decided to determine which miRNAs target murine *Nrg3* mRNAs. Based on the mouse genome browser, the *Nrg3* gene produces three distinct mRNAs, each with a different 3′-UTR length (Figure 2D). We observed that two of these mRNAs have very short 3′-UTRs (56 nts) that do not possess any miRNA binding sites. In contrast, the NM_001190187 mRNA contains a substantially longer 3′-UTR (1582 nts) with five different miRNA binding sites grouped into two clusters (Figure 2E). The miRNAs binding to these sites, i.e., miR-137-3p, miR-155a-5p, miR-190b-5p, miR-204-5p, miR-30b-5p, miR-30c-5p, miR-30d-5p, miR-30e-5p, miR-384-3p, were selected for the correlation analysis with impulsivity. We established that only miR-190b-5p is significantly correlated with impulsive action and that this correlation is negative, meaning that with increased impulsivity of BXD mice the expression of miR-190b-5p in the amygdala is lowered. We further determined correlation of miR-190b-5p with the overrepresented compound traits and only observed significant correlations with two metabolic traits (Table 4, Figure 3).

**Table 4:**
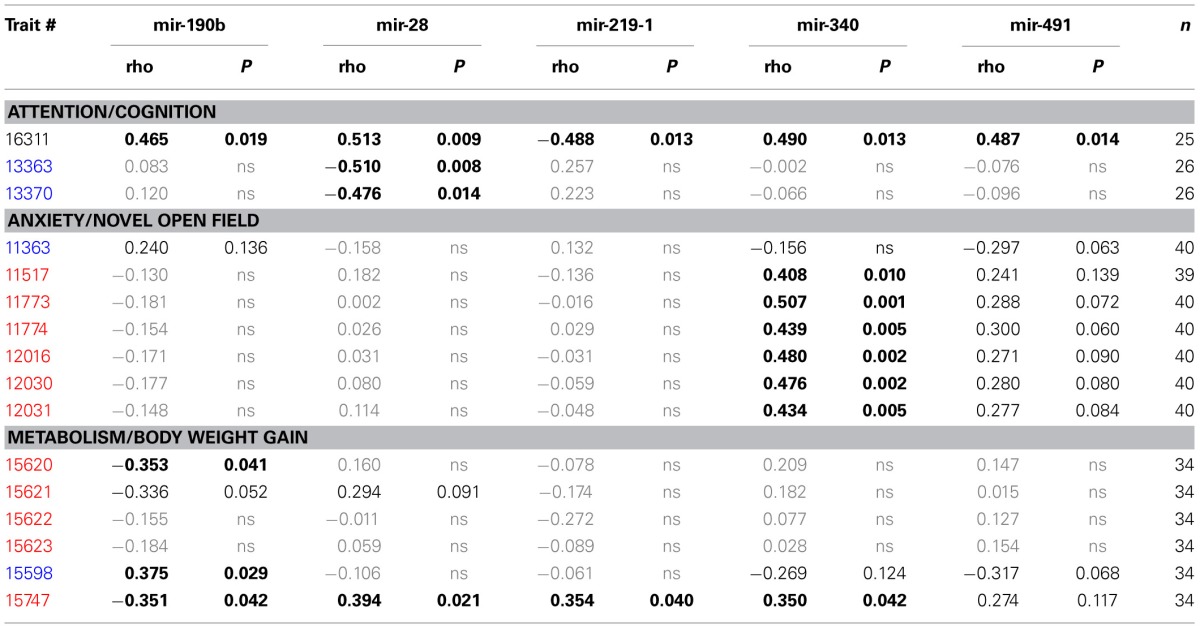
**Correlation between overrepresented compound traits and selected microRNAs expressed in amygdala**.

For three significantly impulsivity-correlated compound traits, the individual traits are shown. In addition, two traits (#15598, #15747) were added to the Body weight gain compound trait, as they relate to body weight and food intake, respectively. Trait numbers correspond to traits are described in Table 1, and trait color indicates the correlation with impulsivity (red, positive; blue negative) Sample rho and P-value of Pearson correlation (n ≥ 25 strains), as well as well as n-number in overlap, with miRNA correlation are shown. P-values < 0.2 are indicated, otherwise non-significant (ns; gray); P-values < 0.05 indicates significant correlation (bold). Traits with n < 25 strains in overlap with miRNA expression are not shown.

Finally, we established that expression of miR-28a-5p, miR-219a-5p, miR-340-5p, and miR-491-5p, although not directly related to the impulsivity locus or *Nrg3*, is significantly correlated with the impulsivity trait (Table 3). Three of these miRNAs (miR-28a-5p, miR-340-5p, and miR-491-5p) were negatively correlated with impulsivity, whereas expression of miR-219a-5p showed a positive correlation. Furthermore, the expression of this set of miRNAs was assessed for correlated expression with the overrepresented compound traits. The expression of miR-340-5p was strongly and positively correlated with all anxiety traits, most of which represented rearing behavior (Table 4, Figure 3). Rearing could probably represent phenotypes of vigilance or learning (Görisch and Schwarting, 2006). Also, expression of miR-491-5p was weakly positively correlated with these rearing traits. Consistent with the positive correlation of miR-28a-5p with impulsivity, it showed a negative correlation with the attention traits of the 5CSRTT (Table 4, Figure 3). However, expression of miR-219a-5p was not correlated with any other trait (Table 4, Figure 3).

### Plausible role of correlated microRNAs in neuronal function

As a next step in identifying putative regulators of impulsivity and overrepresented compound traits in the amygdala, we attempted to better understand their role in neuronal function. Because a single miRNA typically regulates several protein-coding mRNAs we first established which gene products are, in addition to *Nrg3*, miR-190b-5p targets. Subsequently, to determine the Nrg3-related network of miR-190b-5p targets, we predicted biological interactions of these gene products with Nrg3, as well as their functional importance using TargetScanMouse, GeneMania and DAVID (Huang et al., 2007; Friedman et al., 2009; Warde-Farley et al., 2010), respectively.

**Figure 4:**
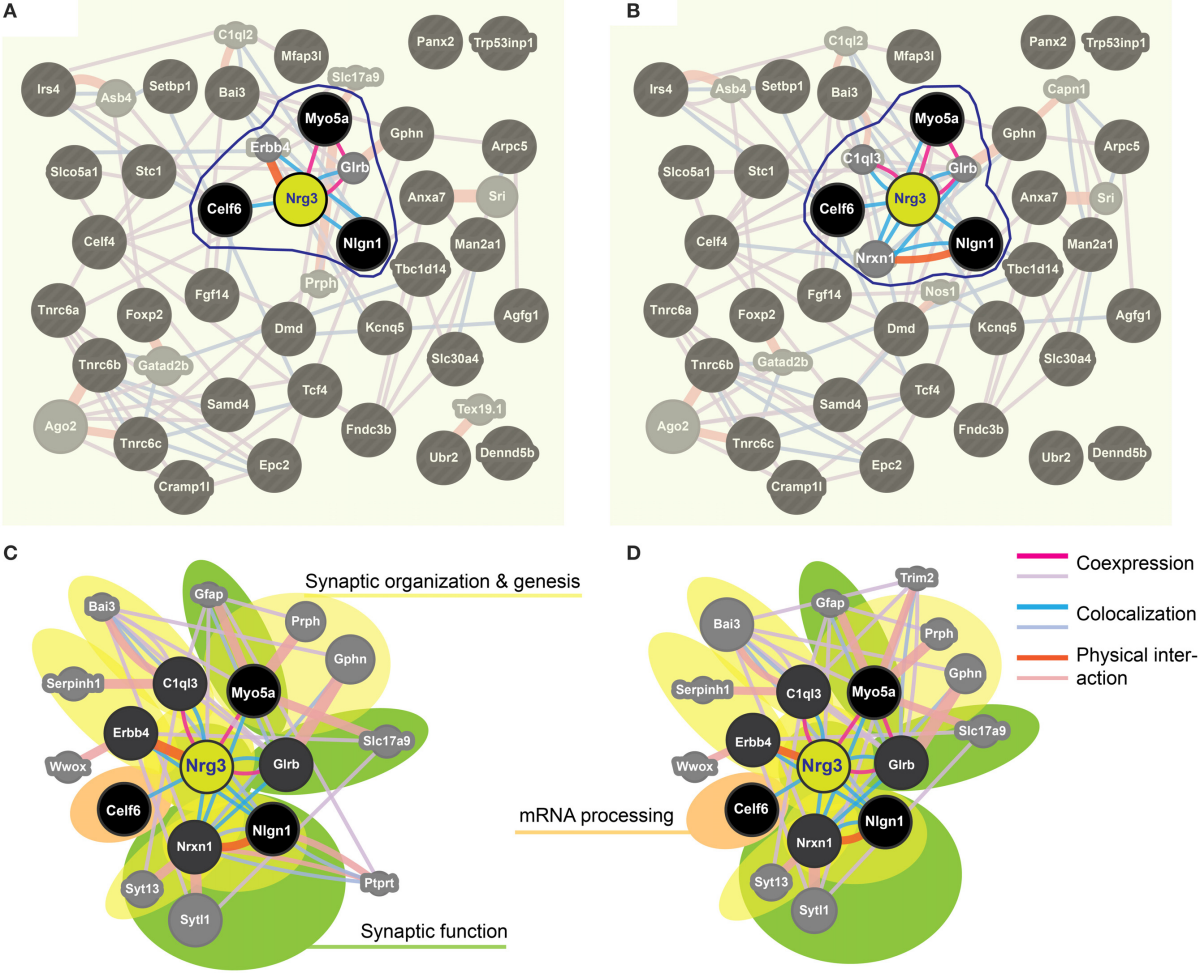
**Impulsivity-related miR-190b and Nrg3 expression network in amygdala. (A,B)** Networks of reciprocal interactions of 34 targets (black) of Nrg3 selected based on GeneMania stringent interaction criteria (for full names see **Table 5**), weighted equally by network (a), or weighted for biological process **(B)**. A maximum of 10 additional genes is shown (gray). Highlighted (encircled) are gene products and their interactions directly linked to Nrg3. **(C,D)** Nrg3 network of closely interacting gene products, with direct miR-190b-5p targets (black) and associated genes (dark gray) (see **A,B**) and their contribution to gene ontology (GO) biological processes in the Nrg3-network (color-coded: yellow, synaptic organization and genesis; green, synaptic function; orange, mRNA processing). A legend describing interaction categories is shown. Link thickness corresponds to strength (weight) of the interaction, and size of shape (gray only) indicates the weight of the interaction in the network.

We applied stringent TargetScan conditions and selected only targets with simultaneously a high aPCT score and a low total context score (top 33% of all targets; see Methodology). This approach, ensuring selection of high probability interactions between miR-190b-5p and its targets, yielded 34 genes (Table 5). In order to view genes that are related to the Nrg3 network, the 34 selected targets underwent an interaction network analysis using GeneMania, in which we only selected gene products directly linked to Nrg3, based on evidence indicating co-localization, co-expression and physical interaction with Nrg3, for different network weighting (Figures 4A,B). Besides three miR-190b-5p targets (Myo5a, Celf6, and Nlgn1) interacting directly with Nrg3, we also discovered additional gene products as members of the Nrg3 network, which were ErbB4, the receptor of Nrg3, and Grlb, the b-subunit of the glycine receptor (Figure 4A). In addition, the adhesion molecule Nrxn1, and the complement component C1ql3 (Figure 4B) were detected. In order to evaluate an Nrg3-network of closely interacting gene products, we took these eight genes for a second round of establishing gene interactions (Figures 4C,D). Relationships of each gene with a particular biological term within each process are shown in Table 6. It appears that this network contributes mostly to three biological processes, all of them pertinent to the neuronal (synaptic) function (Figures 4C,D). Thus, miR-190b-5p, via its targets and genes within the Nrg3-network, seems to control synaptic activity.

**Table 5:** **Targets of miR-190b**.

**#**	**Gene symbol**	**mRNA accession #**	**Gene full name**	**aPCT score**	**TC score**
1	Dennd5b	NM_177192	DENN/MADD domain containing 5B	0.44	−0.42
2	Dmd	NM_007868	Dystrophin, muscular dystrophy	0.44	−0.33
3	Tnrc6b	NM_144812	Trinucleotide repeat containing 6b	0.44	−0.31
4	Kcnq5	NM_001160139	Potassium voltage-gated channel, subfamily Q, member 5	0.44	−0.30
5	Irs4	NM_010572	Insulin receptor substrate 4	0.43	−0.28
6	Trp53inp1	NM_001199105	Transformation related protein 53 inducible nuclear protein 1	0.42	−0.27
7	Epc2	NM_172663	Enhancer of polycomb homolog 2 (Drosophila)	0.40	−0.37
8	Gphn	NM_145965	Gephyrin	0.40	−0.27
9	Celf4	NM_001146292	CUGBP, Elav-like family member 4	0.40	−0.21
10	Fgf14	NM_010201	Fibroblast growth factor 14	0.40	−0.16
11	Setbp1	NM_053099	SET binding protein 1	0.37	−0.26
12	Tnrc6a	NM_144925	Trinucleotide repeat containing 6a	0.35	−0.31
13	Myo5a	NM_010864	myosin 5A	0.30	−0.36
14	Samd4	NM_001037221	Sterile alpha motif domain containing 4	0.30	−0.28
15	Foxp2	NM_053242	Forkhead box P2	0.30	−0.21
16	Tnrc6c	NM_198022	Trinucleotide repeat containing 6C	0.27	−0.22
17	Bai3	NM_175642	Brain-specific angiogenesis inhibitor 3	0.24	−0.34
18	Stc1	NM_009285	Stanniocalcin 1	0.23	−0.45
19	Nlgn1	NM_001163387	Neuroligin 1	0.23	−0.40
20	Arpc5	NM_026369	Actin related protein 2/3 complex, subunit 5	0.22	−0.35
21	Fndc3b	NM_173182	Fibronectin type III domain containing 3B	0.22	−0.25
22	Slco5a1	NM_172841	Solute carrier organic anion transporter family, member 5A1	0.22	−0.24
23	Panx2	NM_001002005	Pannexin 2	0.22	−0.13
24	Slc30a4	NM_011774	solute carrier family 30 (zinc transporter), Member 4	0.22	−0.11
25	Agfg1	NM_010472	ArfGAP with FG repeats 1	0.22	−0.11
26	Mfap3l	NM_001177881	Microfibrillar-associated protein 3-like	0.22	−0.10
27	Anxa7	NM_001110794	Annexin A7	0.22	−0.10
28	Celf6	NM_175235	CUGBP, Elav-like family member 6	0.22	−0.10
29	Ubr2	NM_001177374	Ubiquitin protein ligase E3 component n-recognin 2	0.22	−0.09
30	Cramp1l	NM_020608	Crm, cramped-like (Drosophila)	0.22	−0.09
31	Nrg3	NM_001190187	Neuregulin 3	0.22	−0.08
32	Man2a1	NM_008549	Mannosidase 2, alpha 1	0.22	−0.08
33	Tbc1d14	NM_001113362	TBC1 domain family, member 14	0.21	−0.32
34	Tcf4	NM_001083967	mRNAion factor 4	0.21	−0.24

Targets of miR-190b predicted by TargetScanMouse are shown. Targets were sorted based on the aPCT (>0.2) and total context (TC; <-0.07) scores, which represent the aggregated probability of conserved targeting and predictions based on four features of the putative microRNA-binding site, respectively. For each target, its symbol, a representative mRNA, and full name of the gene from which the target is produced are shown.

**Table 6:**
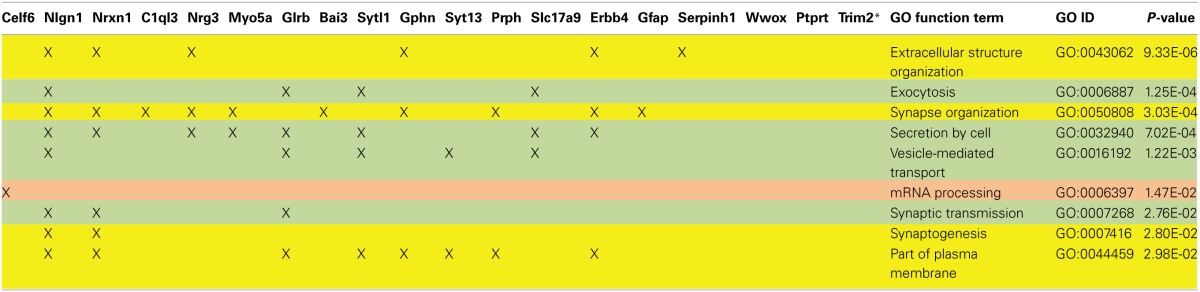
**Characterization of Nrg3-related network and their biological functions**.

A link between each gene product and the gene ontology (GO) biological function term, as determined by GeneMania and confirmed by DAVID and GeneCards, is marked, with function domains color-coded: yellow, synaptic organization and genesis; green, synaptic function; orange, mRNA processing (see also Figures 4C,D). GO IDs and P-values (FDR-corrected) are shown. Celf6, Elav-like family member 6; Nlgn1, neuroligin 1; Nrxn1, neurexin 1; C1ql3, complement C1q-like protein 3; Nrg3, neuregulin 3; Myo5a, myosin5 isoform A; Glrb, glycine receptor, beta subunit; Bai3, brain-specific angiogenesis inhibitor 3; Sytl1, synaptotagmin-like protein 1; Gphn, gephyrin; Syt13, synaptotagmin XIII; Prph, peripherin, Slc17a9, solute carrier family 17 member 9; Erbb4, v-erb-a erythroblastic leukemia viral oncogene homolog 4; Gfap, glial fibrillary acidic protein; Serpinh1, serine (or cysteine) peptidase inhibitor clade H member 1; Wwox, WW domain-containing oxidoreductase; Ptprt, type T receptor of a protein tyrosine phosphatase; Trim2, tripartite motif-containing protein 2. ^*^Although Trim2 localizes to cytoplasmic filaments, its function has not been identified yet. P-value < 0.05 was considered significant.

**Figure 5:**
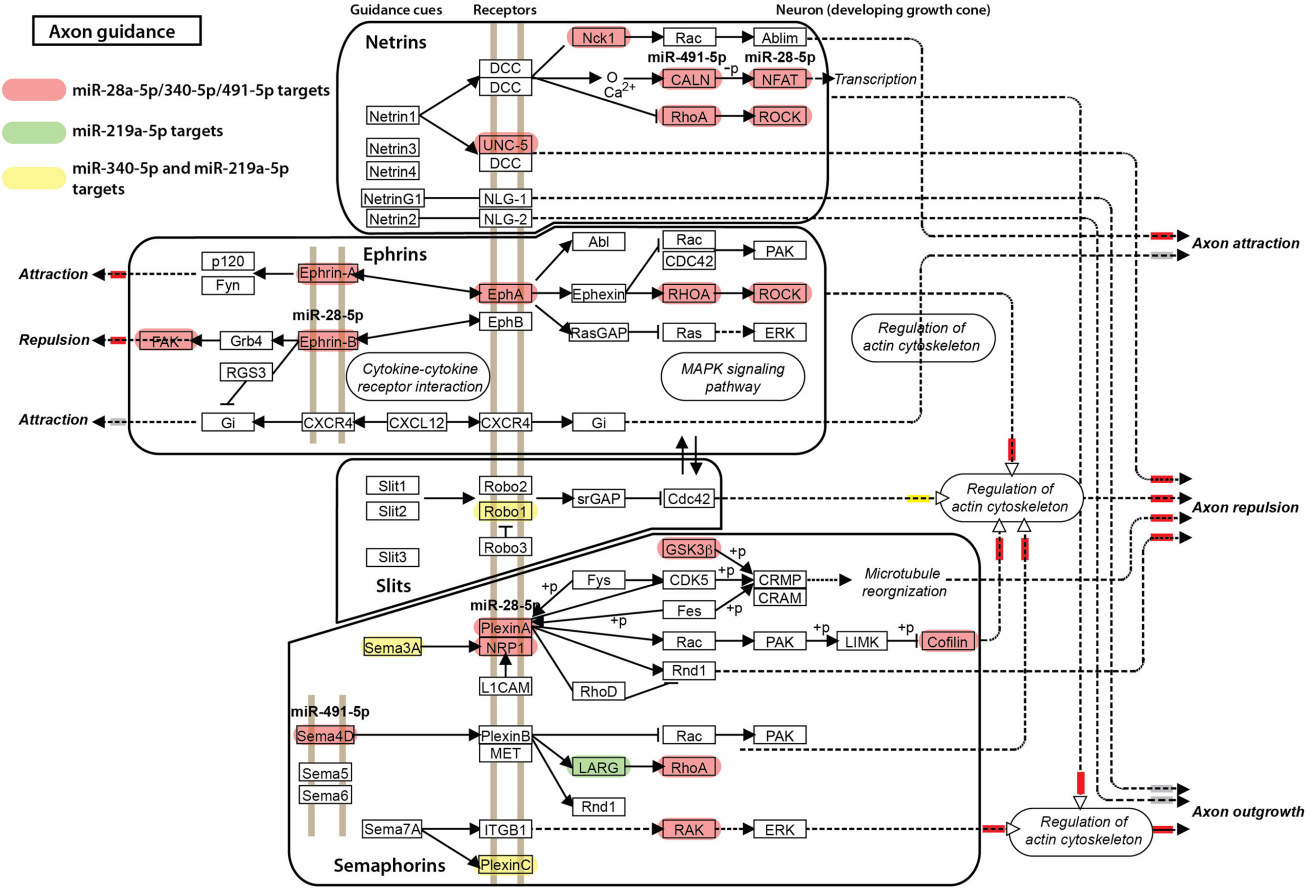
**Axonal guidance KEGG pathway and its regulation by microRNAs correlated with impulsivity**. Gene products targeted by miR-28a-5p, miR-340-5p, and miR-491-5p are shown in red, a miR-219a-5p target in green, and the miR-340-5p/miR-219a-5p targets in yellow. Axonal guidance is broken down into groups controlled by specific guidance cues (netrins, ephrins, slits, and semaphorins) for clarity. The final, putative effects on axonal attraction, repulsion and outgrowth are color-coded: inhibition is depicted by a small red box next to the arrowhead, stimulation by a yellow box, while no effect by a gray box.

For the other miRNAs, miR-28a-5p, miR-219a-5p, miR-340-5p, and miR-491-5p, which were significantly correlated with the impulsivity trait (Table 3), the expression of three (miR-28a-5p, miR-340-5p, and miR-491-5p) was correlated in the same direction (positive correlation) with the impulsivity trait, whereas the expression of miR-219a-5p was correlated in the opposite direction. Typically, miRNAs work as gene product suppressors; increased expression of a miRNA decreases expression of its targets, while decreased expression of a miRNA increases expression of its targets. To understand the contribution of these miRNAs to biological processes, we uploaded all three positively correlated microRNAs as a one group and the negatively correlated microRNA (miR-219a-5p) as a second group into mirPath v2.0 (Vlachos et al., 2012). This program allows for determination of interactions of pathways regulated by multiple miRNAs or a single miRNA. The simultaneous input of all 4 miRNAs, although possible, would make it difficult to distinguish down-regulated from up-regulated effects of miRNAs on their targets. The top five of enriched, neuronal function-related pathways are shown in Table 7 (upper part) with the axonal guidance pathway being regulated most. We next determined in a similar fashion pathways regulated by miR-219a-5p, a brain-specific miRNA. Axonal guidance also appeared to be one of the pathways targeted by miR-219a-5p (Table 7, lower part). Since miR-219a-5p is correlated with impulsivity in an opposite direction as the three other miRNAs, it could attenuate some of the effect of these miRNAs. Figure 5 shows the axonal guidance pathway with gene products targeted by specific miRNAs used in this study. Axonal guidance is controlled by several guidance cues (netrins, ephrins, slits and semaphorins), which affect axonal attraction, repulsion and outgrowth. The effect of miR-219a-5p seems to be small and mainly limited to slits-related guidance cues and (partially) semaphorins cues, whereas the netrins and ephrins cues were affected exclusively by the positively correlated miRNAs (miR-28a-5p, miR-340-5p, and miR-491-5p). Thus, the combinatorial effect of all miRNAs correlated with impulsivity on the KEGG axonal guidance pathway is carried out mostly by the miRNAs that are positively correlated with impulsivity (Figure 5). Considering that positive correlations mean that an increase in impulsivity is associated with an increase in miRNA expression in the amygdala, and since the main action of miRNAs is suppression of its targets, we concluded that the miRNAs correlating with impulsivity most likely attenuate axon repulsion in the amygdala (Figure 5).

**Table 7:** **Biological pathways regulated by microRNAs correlated with impulsivity trait**.

**KEGG biological pathway**	***P*-value**
**miR-28a, miR-340, and miR-491**	
Axon guidance	2.18E-09
Wnt signaling pathway	1.46E-07
Endocytosis	2.38E-07
MAPK signaling pathway	1.28E-05
Focal adhesion	1.40E-05
**miR-219a**	
Endocytosis	1.45E-02
Circadian rhythm	2.81E-02
Axon guidance	3.34E-02
Wnt signaling pathway	4.73E-02

Enriched pathways related to neuronal functions are shown. Contribution of miR-28a-5p, miR-340-5p and miR-491-5p was assessed simultaneously due to their synchronized positive correlation. We have analyzed miR-219a separately because it was the only miRs correlated negatively with impulsivity. Analysis was performed using DIANA mirPath algorithm and KEGG software with FDR correction. P-value < 0.05 was considered significant.

Two other pathways were commonly regulated by both the positively- and the negatively-correlated miRNAs. These were Wnt signaling and endocytosis (Table 7). Similarly to the axonal guidance, both of these pathways seem to be more affected by the positively correlated miRNAs (miR-28a-5p, miR-340-5p, and miR-491-5p) rather then the negatively correlated one (miR-219a-5p), of which the effect on these pathways is barely significant (Table 7).

In summary, our comprehensive bioinformatic analysis seems to indicate that miRNAs in the amygdala may contribute to the development of the impulsivity trait. On one hand, miR-190b-5p seems to control synaptic activity, whereas other miRNAs (primarily miR-340-5p) may control axonal guidance.

## Discussion

Impulsive action, the tendency to act “on a spot” without a foresight and the inability to inhibit such a prepotent response, is a complex behavioral trait. This type of impulsivity can be advantageous in situations requiring a rapid response, but often is detrimental and associated with psychiatric disorders. Impulsivity is moderately heritable (Kuntsi et al., 2006; Schachar et al., 2011; Crosbie et al., 2013), which suggests partial genetic etiology (Bevilacqua and Goldman, 2013). We have recently established the presence of a genetic locus for motor impulsivity in the genetic resource of BXD strains demonstrating an important role for *Nrg3* in the mPFC (Loos et al., 2014).

Here, we observed that in BXD mice the impulsivity trait is significantly associated with traits relevant to psychiatric disorders: attention, depression, metabolism and forms of anxiety. Indeed, impulsive behavior is augmented in several psychiatric disorders, of which the most well-known is ADHD, in which it is one of the DSM-V criteria (American Psychiatric Association). The negative correlations of impulsivity with attention parameters, such as the % of correct responses as found here (Figure 1), are well-known and have been previously observed in different strains or pertubations (Loos et al., 2009; Counotte et al., 2011; Agnoli and Carli, 2012). A concerted, balanced relationship between attention and behavioral inhibition is central to the executive function model and effective adaptation to an ever-changing environment (Bari and Robbins, 2013).

In addition, impulsivity is often observed in bipolar patients, and co-occurs with periods of mania (McElroy et al., 1996; Lombardo et al., 2012). Interestingly, a positive family history of bipolar disorder could indicate an elevated risk to develop bipolar disorder in children with ADHD (Sachs et al., 2000). Many animal models of depression try to capture aspects of the disease in the anxiety domain, with classical anxiety tests, such as response to novelty in an open field or elevated plus maze, and test that assess helplessness and a low effort to escape, such as the tail suspension test and forced swim test (Palanza, 2001; Krishnan and Nestler, 2011; Zhu et al., 2014). Immobility in a tail suspension test (traits #11946, #11432), serving as proxy for depression, was negatively correlated with impulsivity. Although this is of interest from a clinical perspective, motor impulsivity could easily be confused with symptoms of hyperactivity in preclinical models. However, neither in our dataset, nor in the BXD dataset in GeneNetwork, locomotor activity in an open field is correlated with our impulsivity trait. This apparent lack of a correlation between impulsivity and activity in response to novelty has been observed before (Loos et al., 2009). Therefore, the correlation between impulsivity and immobility could indicate a shared biological mechanism.

Whereas motor impulsivity deals with acting before thinking despite possible adverse consequences, compulsivity entails actions that are persistently repeated despite adverse consequences (Robbins et al., 2012). Both psychological constructs have been hypothesized to result from failures of response inhibition or “top-down” cognitive control with overlapping mechanisms and brain areas involved (Wolters et al., 2008; Robbins et al., 2012; Callesen et al., 2013). Compulsivity is conceived as maladaptive behavior contributing to drug-taking, or excessive eating. In this respect, the positive correlation between impulsivity and several metabolism traits (Figure 1) in BXD mice may not come as a surprise. Recent studies showed that impulsivity scores in humans are associated with additive consumption of food and subsequently high BMI (Murphy et al., 2013). This association occurs early in life, as impulsivity is a risk factor for child-obesity (van den Berg et al., 2011; Thamotharan et al., 2013) and obese adolescent individuals have been reported as being more impulsive (Delgado-Rico et al., 2013). This association persists into adulthood (Sutin et al., 2013). Moreover, in psychiatric patients with bipolar disorder the co-occurrence of impulsivity and obesity worsens the prognosis (Galvez et al., 2014).

Since their discovery about 10 years ago, miRNA regulation of gene expression became widely accepted as a fundamental biological process (Farh et al., 2005; Guo et al., 2010). Typically, miRNAs destabilize targeted mRNAs that subsequently undergo degradation. We showed here that expression of miR-190b-5p, but not other miRNAs targeting *Nrg3* mRNAs, is highly correlated with impulsivity. This correlation is negative, which supports a suppressive role of miR-190b-5p on *Nrg3* gene expression. Moreover, expression of miR-190b-5p is negatively correlated with two body mass traits and food intake that significantly correlate with impulsivity (Figure 3). Based on this, we could hypothesize that the shared mechanism between impulsivity and compulsivity could lie in regulation of a miR-190b-5p-directed network.

One of the challenges in understanding miRNA mechanisms is the multiplicity of their actions, in which one miRNA can regulate several targets, and simultaneously a single target can be regulated by several miRNAs. Here, we focused on a selection of the network that is targeted by miR-190b-5p, and that is related to biological relevance of Nrg3. Nrg3 is a part of a complex, intertwined biological network, in which this growth factor can bind and activate Erbb4, a receptor tyrosine kinase (Zhang et al., 1997; Rochat et al., 2008). Erbb4 can upon activation regulate a wide range of biochemical pathways in a cell. Importantly, the expression of *Nrg3* is mainly restricted to the nervous system (Zhang et al., 1997; Evenden, 1999). Mutations in Nrg3 have recently been reported to increase the risk for schizophrenia, and to alter activation of the PFC in humans (Kao et al., 2010; Gupta et al., 2011; Tost et al., 2014). In particular, Nrg3 and ErbB4 could play an important neurodevelopmental role contributing to aberrant cognitive function and social behaviors (Depue et al., 2014; Loos et al., 2014) (Koob, 2009; Koob and Volkow, 2010; Paterson and Law, 2014; Tost et al., 2014). However, Nrg3 has a role beyond this developmental period (Lee et al., 1993; Wightman et al., 1993; Loos et al., 2014), probably maintaining synaptic structures during adulthood. In this respect, the role of miRNAs is important, because the network found for miR-190b-5p targets and associated genes includes those that have a function in synapse formation and stabilization. Recently, miR-190 has been shown to be involved in regulation of synapses by drugs of abuse. Fentanyl (but not morphine) decreases miR-190 levels and thereby regulates NeuroD by binding to its 3′-UTR (Ambros and Lee, 2004; Landgraf et al., 2007; Friedman et al., 2009; Zheng et al., 2010). NeuroD is critical for dendritic spine stability without affecting axon growth (Gaudillière et al., 2004; Siegel et al., 2011). One of the direct targets of the miR-190b-5p network is neuroligin (Nlgn1), which is a key synaptic factor that mediates the formation and maintenance of synapses. It acts as a cell adhesion protein expressed in the post-synaptic compartment, and interacts with ß-neurexins located pre-synaptically. Together, neuroligin and ß-neurexins hold both synaptic compartments close to each other, helping to create a synaptic cleft. Importantly, alterations in expression of neuroligin are linked to psychiatric diseases, specifically autism spectrum disorders (Farh et al., 2005; Lewis et al., 2005; Südhof, 2008).

Besides miR-190b-5p, the expression of miR-340-5p, miR-28a and miR-491 in the amygdala was negatively correlated with impulsivity. Whereas miR-340-5p was detected as downregulated with age in serum (Im and Kenny, 2012; Xu et al., 2012; Nestler, 2014; Zhang et al., 2014), miR-340-5p was upregulated in the hippocampus of FMRP mice (Hébert and De Strooper, 2009; Liu et al., 2014), a neurodevelopmental model characterized by more immature dendritic spines (Bassell and Warren, 2008; Pietrzykowski, 2010). Likewise, in melanocytes, miR-340 is able to increase dendritic formation (Sánchez-Mora et al., 2013; Jian et al., 2014). In this respect, the negative correlation of miR-340-5p with impulsivity could relate to a decrease in synaptic plasticity in the amygdala of impulsive mice.

As yet, miR-28a-5p and miR-491-5p have been mainly studied in cancer, and more specifically in the formation of gliomas (Malzkorn et al., 2010; Németh et al., 2013; Li et al., 2014). Both derivatives of the mir-491 precursor, miR-491-5p and -3p control key hallmarks of glioma carcinogenesis, repressing proliferation (Li et al., 2014; Loos et al., 2014). In addition, in the PFC of depressed patients, as well as in serum of schizophrenic patients, a decrease in miR-491-3p is observed (Cole and Robbins, 1989; Shi et al., 2012; Serafini et al., 2014). Moreover, the expression of miR-219-5p, which was positively correlated with impulsivity, is downregulated in gliomas. This is in concordance with its role in inhibiting proliferation of medulloblastoma cells and migration of glioma cells, as well as promoting neural differentiation (Hudish et al., 2013; Rao et al., 2013; Shi et al., 2014). The role of miR-219 in the brain is limited to being downregulated upon disruption of NMDAR signaling in the PFC, and downregulation of miR-219 inhibits the NMDAR antagonist dizocilpine-induced effect on locomotion and stereotypy (Kocerha et al., 2009). Taken together, although a role for these miRNAs in alterating synaptic plasticity, in part, through the changes in development of spines or dendrites seems likely, this has not been established yet. Based on our results, we propose here that miR219-5p along with miR-340-5p, miR-28a-5p and possibly miR-491-5p could have a key role in the development of psychiatric diseases, possibly by affecting the balance between neuronal outgrowth and differentiation in the context of synapse plasticity, maturation and maintenance.

## Conclusions

Our goal here was to use multiple bioinformatic resources to select miRNAs relevant to impulsivity. Our data suggest that miR-190b-5p is a strong candidate of a biological network regulation centered around Nrg3 in relation to impulsive and compulsive traits. This network could affect synaptic processes in amygdala. On a critical note, these findings stem from big datasets, which also could have larger (technical) variation, and therefore need validation at multiple levels. On the other hand, the strict criteria that we have applied, in combination with the fact that we tested a selective hypothesis should be a more powerful approach reducing false-positives, as suggested before (Chesler et al., 2005), and therefore lead to a specific set of experiments that can be tested in the lab. These could entail performing quantitative gene expression (e.g., real-time PCR) for miRs, Nrg3 and target genes, as well as protein levels for Nrg3 and target genes (e.g., quantitative proteomics), in different BXD strains, or after intervention (e.g., RNA interference) of either levels of Nrg3 (Nrg3 KO, Nrg3 overexpression) or specific miRs. Although we focused here on the amygdala, because of its role in emotional aspects of impulsivity, it is evident that impulsivity stems from the complex interactions of several brain regions, including the striatum and cortical regions (Crews and Boettiger, 2009; Basar et al., 2010; Kerr et al., 2014). Therefore, it will be of great interest to show directly a role of these miRNAs in impulsivity and compulsivity in these brain regions by wet lab experiments, as mentioned above.

Psychiatric disorders are complex amalgamations of behavioral traits rooted in molecular mechanisms. With the emerging role of miRNAs as major regulators of gene and protein expression, and cellular function, they are also attractive targets for therapeutic approaches (Dinan, 2010; Chan and Kocerha, 2012)., We propose here that a set of miRNAs contributes to the regulation of synaptic plasticity in the amygdala, which could bring us a little bit closer to understanding mechanisms in psychiatric disorders and creating new therapeutic options.

### Conflict of interest statement

Sabine Spijker and Andrzej Z.Pietrzykowski received a partial restitution for the travel to the INCF Short Course on Neuroinformatics, Neurogenomics and Brain Disease. The authors declare that the research was conducted in the absence of any commercial or financial relationships that could be construed as a potential conflict of interest.
